# Endocrine Therapy for Hormone Receptor-Positive Advanced Breast Cancer: A Nation-Wide Multicenter Epidemiological Study in China

**DOI:** 10.3389/fonc.2020.599604

**Published:** 2021-02-11

**Authors:** Yun Wu, Yiqun Han, Pei Yu, Quchang Ouyang, Min Yan, Xiaojia Wang, Xichun Hu, Zefei Jiang, Tao Huang, Zhongsheng Tong, Shusen Wang, Yongmei Yin, Hui Li, Runxiang Yang, Huawei Yang, Yuee Teng, Tao Sun, Li Cai, Hongyuan Li, Xi Chen, Jianjun He, Xinlan Liu, Shune Yang, Youlin Qiao, Jinhu Fan, Jiayu Wang, Binghe Xu

**Affiliations:** ^1^ Department of Medical Oncology, National Cancer Center/National Clinical Research Center for Cancer/Cancer Hospital, Chinese Academy of Medical Sciences and Peking Union Medical College, Beijing, China; ^2^ Department of Cancer Epidemiology, National Cancer Center/National Clinical Research Center for Cancer/Cancer Hospital, Chinese Academy of Medical Sciences and Peking Union Medical College, Beijing, China; ^3^ Department of Breast Cancer Medical Oncology, Hunan Cancer Hospital, Changsha, China; ^4^ Department of Breast Disease, Henan Breast Cancer Center, The Affiliated Cancer Hospital of Zhengzhou University & Henan Cancer Hospital, Zhengzhou, China; ^5^ Department of Medical Oncology, Zhejiang Cancer Hospital, Hangzhou, China; ^6^ Department of Medical Oncology, Fudan University Shanghai Cancer Center, Shanghai, China; ^7^ Department of Breast Cancer, The Fifth Medical Centre of Chinese PLA General Hospital, Beijing, China; ^8^ Department of Breast and Thyroid Surgery, Union Hospital, Tongji Medical College, Huazhong University of Science and Technology, Wuhan, China; ^9^ Department of Breast Oncology, Key Laboratory of Breast Cancer Prevention and Therapy, National Clinical Research Center for Cancer, Tianjin Medical University Cancer Institute and Hospital, Tianjin, China; ^10^ Department of Medical Oncology, State Key Laboratory of Oncology in South China, Sun Yat-sen University Cancer Center, Guangzhou, China; ^11^ Department of Medical Oncology, The First Affiliated Hospital of Nanjing Medical University, Nanjing, China; ^12^ Department of Breast Surgery, Sichuan Province Tumor Hospital, Chengdu, China; ^13^ Department of Medical Oncology, Yunnan Cancer Hospital, Kunming Medical University, Kunming, China; ^14^ Department of Breast Surgery, The Affiliated Tumor Hospital of Guangxi Medical University, Guangxi, China; ^15^ Department of Medical Oncology and Thoracic Surgery, The First Hospital of China Medical University, Shenyang, China; ^16^ Department of Medical Oncology, Cancer Hospital of China Medical University, Liaoning Cancer Hospital and Institute, Key Laboratory of Liaoning Breast Cancer Research, Shenyang, China; ^17^ The 4th Department of Internal Medical Oncology, Harbin Medical University Cancer Hospital, Harbin, China; ^18^ Department of the Endocrine and Breast Surgery, The First Affiliated Hospital of Chongqing Medical University, Chongqing Medical University, Chongqing, China; ^19^ Department of Medicine Oncology, 900 Hospital of the Joint Logistics Team, Fuzhou, China; ^20^ Department of Breast Surgery, The First Affiliated Hospital of Xi’an Jiaotong University, Xi’an, China; ^21^ Department of Oncology, General Hospital of Ningxia Medical University, Ningxia, China; ^22^ Department of Breast Cancer and Lymphoma, Affiliated Tumor Hospital of Xinjiang Medical University, Urumqi, China

**Keywords:** advanced breast cancer, endocrine treatment, epidemiological study, hormone receptor-positive, nationwide survey

## Abstract

**Background:**

Clinical guidelines generally recommend endocrine therapy (ET) as first-line treatment of hormone receptor-positive advanced breast cancer (HR+ ABC) whereas chemotherapy (CT) should be considered in the presence of life-threatening disease or limited clinical benefit after three sequential ET regimens. However, it is unclear if real-world clinical practice is in accordance with the current guidelines. This study was to present the real-world treatment patterns and ET regimens among HR+ ABC patients in China.

**Methods:**

Using data from the Nation-wide Multicenter Retrospective Clinical Epidemiology Study of Female Advanced Breast Cancer in China (ClinicalTrials.gov identifier: NCT03047889), we investigated the clinicopathological characteristics, clinical profiles, and treatment patterns of HR+ ABC patients from January 2012 to December 2014.

**Results:**

A total of 2,342 patients with HR+ ABC were included in this study. Our findings revealed that, in comparisons with those receiving initial CT (n = 1445), patients initiated ET (n =402) were significantly older, later recurrent after adjuvant treatment, with a lower rate of visceral involvement and a decreasing quantity of metastatic sites. A total of 1,308 patients received palliative ET while only 18.9% patients (n = 247) reached three lines of ET. Among patients completing more than one line of ET, the median treatment duration was 8 months for the first line, 6 months for the second line, and 3 months for the third line for patients receiving ET. In the advanced setting, the choices of palliative ET regimens were diverse, yet aromatase inhibitor (AI) monotherapy was still the overall mainstay of ET; in contrast, patients were less accessible to everolimus plus AI regimen in this population.

**Conclusions:**

Less than one quarter of patients initiated palliative ET for HR+ ABC in routine clinical practice. Patients who received multi-lines of ET experienced successive shorter durations following each line of therapy. This real-life data provides a solid overview of ET for HR+ ABC from China, indicating unmet need for treatment options that improve the effectiveness of endocrine therapy.

## Introduction

Breast cancer (BC) has become a major concern of public health among Chinese women over the past decades (1). With over 360,000 newly diagnosed cases and nearly 100,000 deaths in 2018, breast cancer is the most common malignancy among Chinese women (2). Reportedly, advanced breast cancer (ABC) occurs in approximately 6–10% of newly diagnosed cases, and only 20% of ABC patients are estimated to survive 5 years after its diagnosis (3–6).

Hormone receptor positive (HR+) breast cancer is the most common subtype of BC, accounting for 70% of all breast cancer population (7), in which endocrine therapy (ET) is the mainstay of treatment for the whole course of disease treatment (8). According to the European Society for Medical Oncology clinical guidelines, the National Comprehensive Cancer Network (NCCN) guidelines, minimally toxic ET is the preferred initial option for HR+ ABC patients with the absence of rapid disease progression, symptomatic visceral disease, or suspected endocrine resistance (9, 10).

Evidence-based guidelines have provided some recommendations for optimizing management of HR+ ABC; however, these protocols may not always be consistently followed by physicians in daily practice. Besides, it also depends on disease characteristics, individual inclinations, personal drug responses, and socioeconomic factors (11). The nation-wide epidemiological study is an effective way to gain a better understanding of the treatment pattern of HR+ ABC patients. Previous studies have assessed ET treatment patterns among breast cancer patients in the adjuvant setting in China (12, 13). However, limited data are available for assessing the use of palliative ET for HR+ ABC patients in routine practice.

In this study, we presented data on realistic treatment patterns in a large, multicenter cohort of patients with HR+ ABC in China. This epidemiological study was to improve the understanding of the current status of ET regimens and the factors that influence palliative ET options in HR+ ABC in China, with the aim of providing evidence for the development of current guidelines and medical policies in addition to giving references to the doctors’ continuing medical education.

## Methods

### Study Design and Information Sources

This study was a nation-wide, multi-center, retrospective, epidemiologic study of ABC from January 01, 2012 to December 31, 2014. A total of 21 hospitals, covering seven geographic regions, were involved in this study, with Cancer Institution and Hospital, Chinese Academy of Medical Sciences (CHCAMS) as the leading center for the overall coordination. This study included pathologically confirmed female ABC inpatients in selected months. In our study, inpatients were defined as patients that were treated in inpatient hospitals, whereas outpatients were patients treated in outpatient cancer care facilities, clinics, and day care hospitals. January and February were excluded from the random selection to reduce the confounding effect of the Spring Festival in China. In each selected month, if inpatient admissions were less than the predetermined numbers in that year, more cases from the neighboring months were enrolled. This study was approved by the Ethics Committee of CHCAMS. Patient consent for this study was not required as there were no anticipated risks to the enrolled patients. All data included in this analysis was in aggregate, de-identified information.

### Data Collection and Quality Control

A questionnaire was designed to obtain demographic information and clinical variables, such as age at diagnosis, geographic region, histology grade, hormone receptor (HR), human epidermal growth factor receptor 2 (HER2) status, and treatment information including breast surgery, adjuvant therapy, targeted therapy, radiation therapy, and palliative systemic treatment.

All of the aforementioned information was extracted from medical charts to the designed case report form (CRF) by trained doctors. Then, data was double-entered into computer-based database (Epidata) by two independent data input clerks. All completed databases were sent to CHCAMS for validation. Inconsistencies between the two databases were reported to the local doctors for revision until the databases were consistent. After that, one of the databases was selected to perform a final assessment with the original medical record, then 5% of the medical charts were sent to CHCAMS for quality control review.

### Patient Selection

From the program, we obtained clinical and demographic data on 3,913 ABC patients between 2012 and 2014. The HR and HER2 status were mainly dependent on pathology results in surgery, and patients who had not undergone the surgery included puncture results instead. Study protocols recommended to assess estrogen receptor (ER) and progesterone receptor (PR) status as positive if ≥1% were stained. Any ER or PR positive was regarded as HR positive. When the expression status of primary tumor and metastatic tumor differed, either HR-positive was identified as HR-positive tumor. Therapy lines were documented in the given order, starting with the first therapy given for ABC. Each therapy which started thereafter was considered as a later therapy line regardless of the reason why the previous therapy was terminated. In this research, patients in each group receiving certain treatment were identified as the specified treatment approach alone or in combination with other approaches (for example, the group ‘endocrine therapy’ comprises patients receiving endocrine therapy alone plus other combinations which included endocrine therapy plus HER2-targeted therapy).

### Statistical Analysis

Descriptive analyses were used to characterize the clinicopathologic status of HR+ ABC patients. Descriptive analyses of the number of lines of palliative ET treatment and the number of ET regimens used were also conducted for the HR-positive patients. These patients were divided into two groups: those whose first treatment after ABC diagnosis was ET and those whose first treatment included CT. Baseline characteristics between patients with initial CT and initial ET were compared using χ2 tests for categorical variables, while t-test for continuous variables with normal distribution and Mann–Whitney U test for abnormally distributed variables. Treatment durations were described using medians and interquartile ranges (IQRs). All analyses were performed using SPSS software (version 22.0). A two-sided P value <0.05 was considered to be statistically significant.

## Results

### Characteristics of HR+ Advanced Breast Cancer Cohort by Age Groups

The study population for this analysis consisted of 2,342 HR+ ABC patients, with the median age of 49 years (ranging from 21 to 88 years) and a mean age of 48.97 ( ± 10.08) years. Among the study population, 1,364 (58.2%) patients were diagnosed below 50 years and 978 (41.8%) patients over 50 years. **Table 1** summarized the frequency and proportion of some characteristics of these patient groups. There were a series of significant differences among the cohorts of patient samples including PgR status, bone-only metastasis, and visceral metastasis (P < 0.05). It was noticed that younger patients had obviously higher rate of PgR positive (83.2 *vs*.77.5%, P = 0.002) and bone-only metastasis (28.0 *vs* 23.5%, P = 0.015) in our study. On the contrary, elder group tended to have visceral metastasis than younger patients (47.0 *vs* 42.4%, P = 0.028). However, there were no significant differences in histological grade, ER status, HER2 status, metastases-free interval (MFI), brain metastasis, and number of metastatic sites among the two age groups (P > 0.05). As the leading composition of adjuvant treatment, adjuvant ET was applied to 1,882 (80.4%) patients, of which 976 (51.9%) received selective estrogen receptor modulators (SERMs) and 261 (13.9%) received aromatase inhibitors (AIs).

**Table 1:** Characteristics of patients with HR+ ABC by age group.

Characteristics	Total	Age ≤50 years	Age >50years	*P* value
	(N = 2,342)	(N = 1,364)	(N = 978)	
		N (%)	N (%)	
**Histological grade of primary tumor**				0.73^b^
Grade 1	42 (4.0)	25 (4.0)	17 (4.1)	
Grade 2	668 (64.3)	399 (63.4)	269 (65.6)	
Grade 3	329 (31.7)	205 (32.6)	124 (30.2)	
Unknown	1303	735	568	
**ER status**				0.59^b^
ER+	1958 (91.0)	1145 (91.3)	813 (90.6)	
ER-	193 (9.0)	109 (8.7)	84 (9.4)	
Unknown	191	110	81	
**PgR status**				
PgR+	1618 (80.8)	978 (83.2)	640 (77.5)	0.002*^b^
PgR-	384 (19.2)	198 (16.8)	186 (22.5)	
Unknown	340	188	152	
**HER2 status**				0.88^b^
Positive	718 (37.6)	427 (37.5)	291 (37.8)	
Negative	1192 (62.4)	713 (62.5)	479 (62.2)	
Unknown	432	224	208	
**Metastases-free interval (MFI)**				0.32^b^
≤24 months	1023 (44.1)	609 (44.9)	414 (42.9)	
>24 months	1298 (55.9)	746 (55.1)	552 (57.1)	
Unknown	21	9	12	
**Metastatic sites**				
Bone-only	612 (26.1)	382 (28.0)	230 (23.5)	0.015*^b^
Visceral	1039 (44.4)	579 (42.4)	460 (47.0)	0.028*^b^
Brain	69 (3.0)	47 (3.5)	22 (2.3)	0.093^b^
**Number of metastatic sites**				0.105^b^
1 or 2	1804 (78.6)	1040 (77.4)	764 (80.2)	
≥3	491 (21.4)	303 (22.6)	188 (19.7)	
Unknown	47	21	26	
**Initial palliative treatment**				0.025*^b^
CT	1445 (78.2)	872 (80.1)	573 (75.6)	
ET	402 (21.8)	217 (19.9)	185 (24.4)	
Unknown	495	275	220	
**Palliative ET Lines**				
1st	1308 (55.8)	769 (58.8)	539 (55.1)	0.55^b^
2nd	612 (26.1)	373 (27.3)	239 (24.4)	0.11^b^
3rd	247 (10.5)	157 (11.5)	90 (9.2)	0.07^b^
Average line	2.5	2.58	2.39	0.093^a^

ER, estrogen receptor; PgR, progesterone receptor; HER2, human epidermal growth factor receptor 2; MFI, metastases-free interval.

a t-test.

b Chi-square test.

*P values indicate statistical significance.

### Association Between Disease-Related Factors and First-Line Palliative Treatment Choice

The baseline characteristics of patients according to first-line palliative treatment were summarized in **Table 2**. From the 1,847 patients treated with palliative systemic therapy, 1,445 patients were treated with initial CT, and 402 patients started initial therapy with ET. The mean age of women treated with first-line ET and CT was 48.3 and 50.5 years, respectively (P < 0.0001). Interestingly, significant geographic differences were detected in the CT choice in which the rate of CT performance was higher in the central and south-west area (P < 0.0001). Patients with risk characteristics, such as disease recurrence within 24 months (P = 0.026), increasing number of metastatic sites (P < 0.0001), and visceral involvement (P < 0.0001), generally received palliative CT more often than ET. When looking at HR+ ABC patients categorized by HER2 status, patients with HER2 negative breast cancer were more likely to receive initial ET compared with HER2 positive patients (23.3 *vs* 18.7%, P = 0.038) (**Supplementary Figure 1**). Patients with bone-only metastasis were more likely to receive initial ET compared with patients with visceral metastases, of which 165 (34.0%) patients with bone-only metastases received ET as initial metastatic site, yet 742 (82.9%) of patients with visceral metastases were treated with initial CT (P < 0.0001). Target therapy was administered in 20.7% of patients treated with initial CT compared to 15.1% of patients with initial ET (P = 0.013). Instead, the patients who received the first-line ET had a higher proportion of patients who had previously received adjuvant ET, adjuvant CT, radiotherapy and surgery. There were no statistically significant differences between histological grade (P = 0.16), ER status (P = 0.09), PR status (P = 0.21) and brain metastases (P = 0.305).

**Table 2:** Characteristics of patients with HR+ ABC, according to initial palliative therapy.

Characteristics	Initial chemotherapy	Initial endocrine therapy	*P* value
	(N = 1445)	(N = 402)	
	N (%)	N (%)	
**Age at diagnosis ABC**			
Mean ± SD	48.27 ± 9.61	50.51 ± 11.08	<0.0001*^a^
≤50 years	872 (60.3)	217 (54.0)	0.025*^b^
>50years	573 (39.7)	185 (46.0)	
**Geographic regions**			<0.0001*^b^
North	321 (22.2)	98 (24.4)	
Central	429 (29.7)	90 (22.4)	
South	151 (10.4)	44 (10.9)	
East	250 (17.3)	74 (18.4)	
North-east	109 (7.5)	49 (12.2)	
North-west	104 (7.2)	38 (9.5)	
South-West	81 (5.6)	9 (2.2)	
**Histological grade of primary tumor**			0.16^b^
Grade 1	22 (3.5)	10 (5.7)	
Grade 2	384 (61.9)	115 (66.1)	
Grade 3	214 (34.5)	49 (28.2)	
Unknown	825	228	
**ER status**			0.093^b^
ER+	1200 (91.3)	343 (94.0)	
ER-	115 (8.7)	22 (6.0)	
Unknown	130	37	
**PgR status**			
PgR+	973 (80.3)	287 (83.4)	0.213
PgR-	239 (19.7)	57 (16.6)	
Unknown	233	58	
**HER2 status**			0.038*^b^
Positive	438 (37.0)	101 (30.8)	
Negative	746 (63.0)	227 (69.2)	
Unknown	261	74	
**Metastases-free interval (MFI)**			0.026*^b^
≤24 months	618 (43.1)	148 (36.9)	
>24 months	815 (56.9)	253 (63.1)	
Unknown	12	4	
**Breast surgery**			0.002*^b^
Yes	1308 (91.7)	383 (96.2)	
No	119 (8.3)	15 (3.8)	
Unknown	18	4	
**Radiation therapy**			0.034*^b^
Yes	541 (38.7)	174 (44.6)	
No	858 (61.3)	216 (55.4)	
Unknown	46	12	
**Adjuvant therapy**			
Endocrine therapy	871 (62.1)	280 (73.1)	<0.0001*^b^
Chemotherapy	1156 (81.9)	342 (87.0)	0.016*^b^
**Targeted therapy**			0.013*^b^
Yes	292 (20.7)	59 (15.1)	
No	1120 (79.3)	332 (84.9)	
**Metastatic sites**			
Bone-only	320 (22.1)	165 (41.0)	<0.0001*^b^
Visceral	724 (51.9)	153 (38.4)	<0.0001*^b^
Brain	37 (2.6)	14 (3.5)	0.305^b^
**Number of metastatic sites**			<0.0001*^b^
1	712 (49.9)	245 (61.6)	
2	376 (26.3)	93 (23.4)	
≥3	340 (23.8)	60 (15.1)	
Unknown	17	4	

ABC, advanced breast cancer; SD, standard deviation; ER, estrogen receptor; PgR, progesterone receptor; HER2, human epidermal growth factor receptor 2; MFI, metastases-free interval.

a t-test.

b Chi-square test.

*P values indicate statistical significance.

### Patterns of Treatment

**Figure 1:**
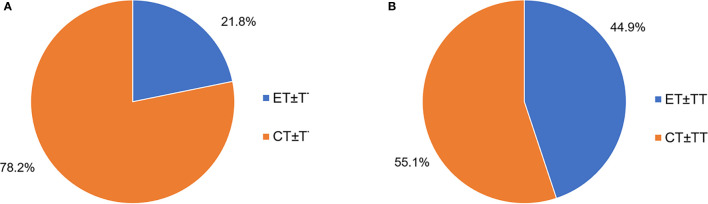
**(A)** First-line palliative treatment options for HR+ ABC patients (N = 1,847). **(B)** Second-line palliative treatment HR+ABC patients (N = 1,424). *ET*, endocrine therapy; *CT*, chemotherapy; *TT*, target therapy.

Following ABC diagnosis, among the patients with first-line treatment information complete (N = 1,847), the majority of patients (78.2%) received CT as the first-line treatment, and 21.8% initiated ET (including endocrine monotherapy, and endocrine combined targeted therapy) at the first line (**Figure 1A**). For patients receiving second-line treatment (N = 1,424), ET took up 44.9% proportion, while CT was still the most common treatment pattern (55.1%) (**Figure 1B**). However, there was no difference in palliative ET Lines between younger and elder patients (**Table 1**).

### Endocrine Treatment Duration

The majority of patients (n = 1,308) received one line of palliative ET during the study period. Among this part of patients, 46.8% (n = 612) received two lines of palliative ET and 18.9% (n = 247) received three lines of therapy. Overall, patients were treated with an average of 2.50 lines of ET (2.58 lines in patients aged ≤50 years *vs* 2.39 lines in >50 years groups) during the follow-up period. The median duration on each line of ET among patients was 8 (3–17) months of first line, dropping to 6 (2–12) months of second line, and 3 (2–8) months of third line (**Table 3**).

**Table 3:** Endocrine therapy regimens and duration time for patients with different treatment line.

Age at diagnosis ABC	1st line			2nd line			3rd line		
	Age ≤50 years (N = 769)	Age >50 years (N = 539)	Total (N = 1,308)	Age ≤50 years (N = 373)	Age >50 years (N = 239)	Total (N = 612)	Age ≤50 years (N = 157)	Age >50 years (N = 90)	Total (N = 247)
**Regimens N (%)**		**N (%)**			**N (%)**			**N (%)**	
SERMs^a^ monotherapy	216 (28.1)	56 (7.0)	272 (20.8)	41 (11.0)	25 (10.5)	66 (10.8)	19 (9.6)	16 (17.8)	35 (14.2)
AIs^b^ monotherapy	215 (28.0)	422 (54.9)	637 (48.7)	155 (41.5)	152 (63.6)	307 (50.2)	56 (35.7)	36 (40.0)	92 (37.2)
SERMs + OFS^c^	37 (4.8)	3 (0.4)	40 (3.1)	8 (2.1)	1 (0.4)	9 (1.5)	5 (3.2)	0 (0)	5 (2.0)
AIs + OFS	253 (32.9)	20 (2.6)	273 (20.9)	107 (28.7)	5 (2.1)	112 (18.3)	31 (19.7)	2 (2.2)	33 (13.4)
Fulvestrant ± OFS	21 (2.7)	24 (3.1)	45 (3.4)	31 (8.3)	25 (10.5)	56 (9.2)	22 (14.0)	15 (16.7)	37 (15.0)
mTOR^d^ + AIs	0 (0)	7 (1.3)	7 (0.5)	6 (1.6)	12 (5)	18 (2.9)	15 (9.6)	6 (6.7)	21 (8.5)
Others^e^	37 (4.8)	9 (1.7)	46 (2.5)	28 (7.5)	19 (8.0)	47 (7.6)	14 (8.9)	15 (16.7)	29 (11.7)
			**Months (IQR)**			**Months (IQR)**			**Months(IQR)**
**Median duration time**			8 (3–17)			6 (2–12)			3 (2–8)

SERMs, selective estrogen receptor modulators; AIs, aromatase inhibitors; OFS, ovarian function suppression; mTOR, mammalian target of rapamycin; IQR, interquartile ranges.

a SERMS include tamoxifen and toremifene.

b AIs include letrozole, anastrozole and exemestane.

c OFS include goserelin, leupraline and surgical castration.

d mTOR include everolimus.

e Others include progestins, OFS monotherapy, combination therapies (SERMs + mTOR, AIs + progestin and fulvestrant + AIs).

### Endocrine Regimens

**Figure 2:**
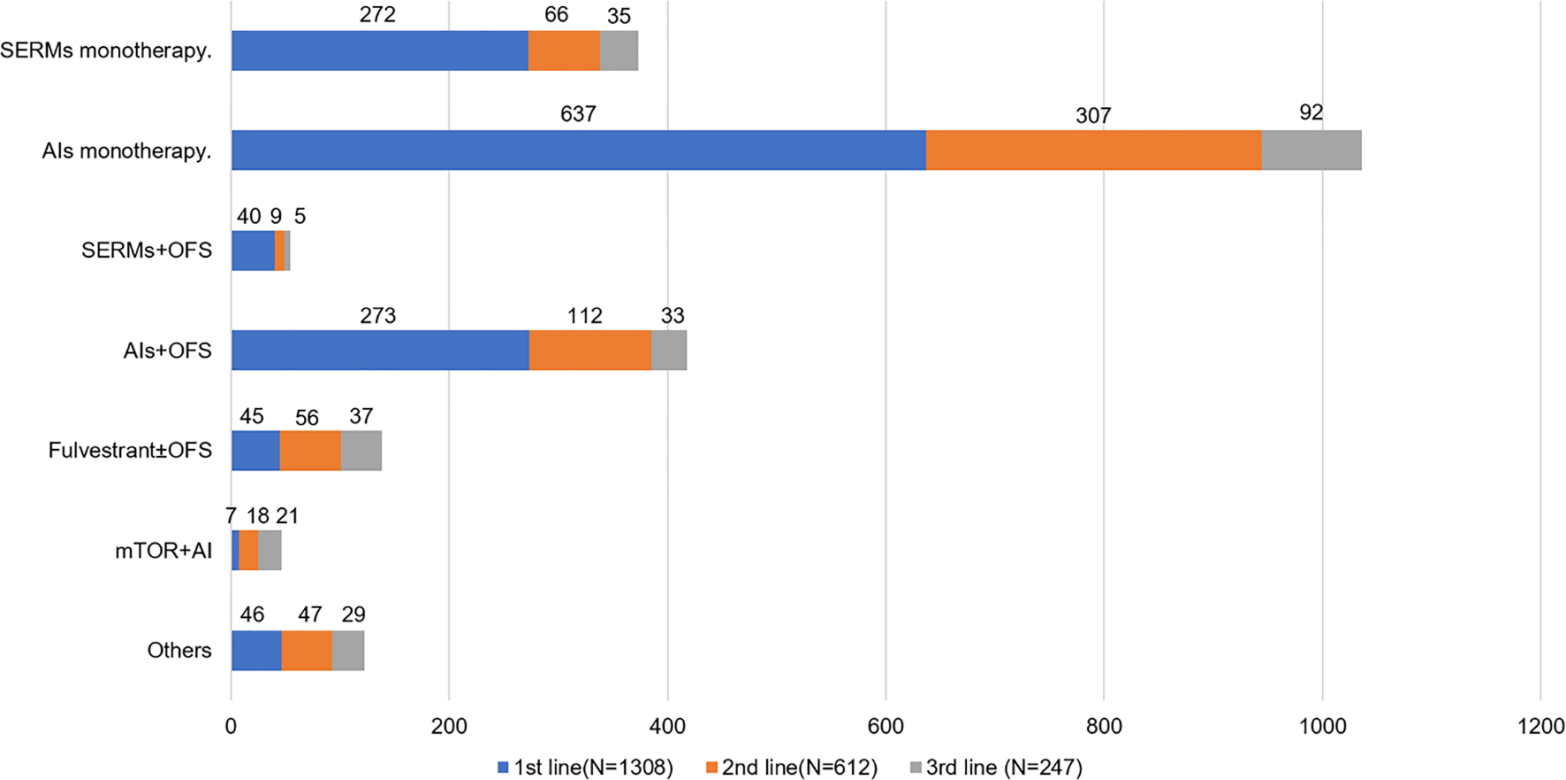
Endocrine regimens use by line for HR+ ABC patients. *SERMs*, selective estrogen receptor modulators; *AIs*, aromatase inhibitors; *OFS*, ovarian function suppression; *mTOR*, mammalian target of rapamycin.

Among patients receiving palliative ET (n = 1,308), AI monotherapy was the most frequent application in the first-line therapy (**Table 3**), of which 637 patients were treated with AI monotherapy in the first line. According to the age group, AI plus OFS was most commonly used in patients aged ≤50 years in the first line (32.9%), followed by SERM monotherapy (28.1%) (**Supplementary Figure 2**). In the second and third lines of ET, AI monotherapy remained in the same dominant position both in >50 and ≤50 years groups, with 50.2 and 37.2% of patients accessible, respectively. Patients aged >50 years were more commonly treated with AI monotherapy than the ≤50 years group in all lines of ET. SERM monotherapy was used as first-line therapy in 272 (20.8%) patients, 10.8% in the second line and 14.2% in the third line. A total of 138 patients (10.6% of the total palliative ET population) were treated with fulvestrant. Regarding the entire patients treated with fulvestrant, 45 (32.6%) were treated in the first line, 56 (40.6%) in the second line, and 37 (26.8%) in the third line. As for ovarian function suppression (OFS) combination therapy (including OFS plus AIs or OFS plus SERMs), there were 313 (23.9%) patients who received this regimen in the first line. The proportion of OFS combination therapy fell off in the second and third lines, which was 19.8 and 15.4% respectively. More patients aged ≤50 years received OFS combination treatment than >50 patients (32.3 *vs* 3.2%). In our study population, a total of 46 (3.5%) patients were treated with everolimus plus AI regimen (**Figure 2**).

## Discussion

This study presented comprehensive information on patient characteristics, initial palliative treatment choice, and ET regimens in routine clinical practice for patients with HR+ ABC in China. Our results clarified that this was a heterogeneous group of patients in terms of treatment administered, and that treatment modalities differed by clinical and demographic variables.

All patients included in our study were ethnically Chinese, of whom the clinicopathological characteristics were significantly different from those of the women in western countries. The mean age at diagnosis of HR+ ABC in China was 48.97 years which was about a decade earlier than that what is reported in the USA and European Union (14, 15). A similar tendency in the age was also found in early breast cancer between Chinese and Western Caucasian women due to racial differences in genetics and lifestyle (13, 16). Population-based study utilizing the SEER database has shown that higher proportion of patients below 50 years had bone-only metastasis than patients over 50 years; however, no significant differences in brain metastasis (17), which were in accordance with the findings of our study. In the current study, 1,882 (80.4%) patients received adjuvant ET for HR positive patients, which is consistent with the previously reported rates (50–86%) (12, 18–22).

An important finding of this large real-life cohort is the high percentage of initial palliative CT. Previous studies have reported a chemotherapeutic delivery in the first line metastatic setting in 24–54% in Europe and America (14, 23–25). However, our study observed that a substantial proportion (78.2%) of patients with HR+ ABC received CT in the first-line setting in China, which was much higher than the percentages of initial CT found in other real-life reports (4, 14, 23–25). Overall, the majority of HR+ ABC patients were not treated with initiated palliative ET, as recommended by the guidelines for patients without the need for immediate tumor reduction (10). It seems that the efficacy of CT is overestimated in real life, and the usage of ET first has not been adopted by a majority of oncologists in China (26).

In this observational study, we found that a group of demographic and clinicopathological features could have an impact on the selection of CT instead of ET as first line palliative treatment. We observed that ET was more extensively used in the elderly compared with younger patients. This tendency fitted in well into other existing studies (14, 24). However, chemotherapy was still a major treatment choice in both younger and older patients. Interestingly, significant geographic differences were detected in the ET choice which was lower in the central and south-west area. The possible explanation for this observation is the disparity of the economic level across the nation. In China, a large proportion of the costs of ET are paid by inpatients and outpatients; thus, patients and physicians’ decisions on whether ET should be used in the first line are affected by the family incomes. Regions such as Northern, Southern, and Eastern China are more economically developed, and people there have higher incomes, and then spend more on health care. Moreover, to achieve the expected efficacy, the patients with history of radiotherapy, surgery, and adjuvant therapy are more inclined to ET in the first line treatment.

We also noted that HER2 status influence the therapeutic decision. Patients with HER2 positive breast cancer were more likely to receive palliative CT compared with HER2 negative patients. Few studies have directly compared ET plus HER2-targeted therapy with CT plus HER2-targeted therapy for HR+ HER2+ ABC patients. The combination of CT and HER2-targeted therapy was shown to present an overall survival (OS) benefit (27), while the treatment based on the combination of ET and HER2-targeted therapy was divergent (28). Therefore, CT plus HER2-targeted therapy was strongly recommended for patients with HR-positive and HER2-positive diseases (9, 10). Additionally, our study showed that visceral metastasis and high tumor burden (≥3 organ metastases) were also significant factors for tailoring CT-first treatment. In contrast, when patients presented with bone-only metastasis, the ratio of ET-first (34%) was increased but was still lower than that in European countries (24). In the previous literature, HR+ ABC patients with bone-only metastasis are recognized with a well-defined prognosis (24, 29, 30) and are suggested to undergo ET-first in a series of guidelines (9, 24, 31). These findings fitted in well into existing studies. Lobbezoo et al. (24) observed in their retrospective analysis that a young-onset age at diagnosis and prior adjuvant ET performance exerted a substantial effect on the CT-first decision; and that bone metastasis conversely tend to ET-first approach. Additionally, Watanabe and colleagues (32) reported that patients with visceral involvement, liver metastasis, ≥3 organ involvement, or primary ABC at diagnosis showed a significantly higher tendency to be allocated to the CT-first group.

There were a number of potential reasons for the low use of initial palliative ET observed in our analysis, in which the leading reason is the endocrine resistance. Notably, the majority of HR+ ABC patients (80.4%) in our analysis have received adjuvant ET for early disease. The increased use of endocrine approaches in patients with HR+ tumors in the adjuvant setting, in particular the use of AIs, enables endocrine treatment resistance as a common challenge in HR+ ABC (33, 34). Also, our study indicated that a decreasing proportion of patients with early recurrence (MFI ≤24 months) chose ET in the first line setting compared with those with late recurrence (MFI >24 months). This might be the result of endocrine resistance which tends to occur in early recurrent patients, as recurrent patients who develop endocrine resistance in primary disease are inclined to respond unsatisfactorily to ET and progress to ABC with a decreasing duration. Thus, current recommendations suggest that patients with evidence of ET resistance should be offered CT (9), which may partly account for the extensive use of CT for HR+ ABC.

The NCCN guidelines for ABC recommend a target of at least three lines of ET conditional on patient’s clinical benefit (10). However, among the patients who received palliative ET in our study, the majority (55.8%) received only one line of ET, and the minority (18.9%) reached three lines of ET. Besides, the treatment duration significantly decreased from the first line onwards. A retrospective study to describe treatment patterns among postmenopausal HR+ metastatic breast cancer (MBC) patients in United States showed that the median treatment duration of ET was 11.6 months in the first line and fell to 4.9 months in the subsequent lines, and fewer than 10% of patients received three or more lines of ET before initiating CT (35). The reason for the low utilization of ET for ABC, especially dropping remarkably in the second- and third-lines, was probably because physicians expect further lines of ET to lack effectiveness due to the development of endocrine resistance eventually (35). Alternatively, patients may rapidly develop progressive disease such as life-threatening visceral metastases after the first-line treatment, making them potential candidates for chemotherapy. This might be the reason for the difficulties in achieving guideline-recommended lines of ET in real-world clinical practice. However, these speculations extend beyond the current study, and the relevant data regarding physicians’ motivations for therapy transformation were not collected. Further research is needed to discuss whether the failure to reach three or more lines of ET could have an impact on OS.

As for ET regimens, we found that the majority of patients received AI monotherapy as total lines of ET; in contrast, patients were less likely to receive everolimus in combination with AIs for the ET regimens of ABC. Over the recent decades, the third-generation AIs such as letrozole, anastrozole, and exemestane have been consistently proven to be superior to tamoxifen both in advanced and in early breast cancer (36). Following AIs, fulvestrant was the next endocrine therapy to be launched in 2010 in China. The FALCON study demonstrated favorable progression-free survival (PFS) in the fulvestrant *versus* anastrozole (37), which has led to approvals for fulvestrant being licensed as first-line ET in postmenopausal HR+ ABC. In the real-life setting, only 138 patients (10.6% of the total ET population) were treated with fulvestrant, and a majority of them were treated in the first and second lines. Data presented here concerning the use of fulvestrant seem to be rather lower than other available data. Hartkopf et al. (15) found that 34.1% were treated with fulvestrant as single agent therapy among 958 patients with HR+ HER2-MBC in German. This diversity probably resulted from the relatively higher price of fulvestrant and limitation of the health insurance.

In addition to the guidelines for ABC, country-specific differences in accessibility may influence the treatment regimen introduced to individualized therapeutics. It was the unprecedented data about the use of everolimus after the approval in China in 2013. Only 3.5% of patients received everolimus combined therapy for the palliative ET, which may be due to the shorter time to market; thus some physicians may not be better educated on the benefits and usages of this drug. The selection of advanced endocrine drugs also includes SERMs, progestins, and so on. Considering the accessibility to drugs in clinical practice in China, although lacking of evidence of large-scale clinical RCTs, it can be used depending on the comprehensive situation of patients.

On the basis of current data, ET agents with the addition of OFS are the recommended option for first-line treatment of premenopausal HR+ advanced women. Due to the lack of updated menstrual status data, we cannot figure out the exact proportion of premenopausal patients using OFS. However, previous literature reported that the median age at natural menopause was 50 years based on 13,406 postmenopausal women in China (38). Therefore, we decided to undertake subgroup analyses of ET regimens taking into account the age as a reference. We found that higher rate of patients aged ≤50 years received OFS combination treatment than >50 patients (32.3 *vs* 3.2%). And there are 37.7% of patients received OFS combination therapy in the first line while 30.8 and 22.9% in the second and third lines for patients aged ≤50 years. However, among premenopausal women in the US, OFS combination therapy was used as first-line therapy in 15.9% patients, 47.8% in the second line and 27.7% in the third line for patients with HR+HER2-ABC (39).

Lack of access to new drugs and drug reimbursement policies in China also limits systemic treatment options for advanced disease. Our findings will provide reference for anti-tumor novel drug research and development, CFDA reform, and continuing medical education of doctors. We were pleased to notice that with the reform of CFDA and health care policies, China has accelerated the approval of new drugs, and the availability of new drugs has significantly improved (40). However, in the context of the presented period and healthcare policy, the group of novel agents including CDK4/6 inhibitors, PI3K inhibitors, and other new drugs have not been used in clinical practice. We hope that these situations will be improved in the upcoming epidemiological survey.

There were some limitations to our study. First, given the retrospective nature of observational study, data may be more likely to be missing or have errors compared with prospectively collected data. For example, missing data that could affect treatment decisions, such as updated menstrual status, could bias the perception of endocrine regimen selection. Second, the data in this study highly depended on the medical record of inpatients, and we did not include the outpatients which may lead to lower proportion of ET usage since a number of patients received ET in the outpatient department. Third, not only age, geographic regions, and tumor characteristics but also performance status, laboratory results, and preferences of patient and doctor are taken into consideration in making clinical treatment decisions. These factors were not accounted for in this study. Additionally, due to the very nature of our study design, survival information of this cohort was unavailable in this retrospective, observational, epidemiological study for prognostic evaluation. Further studies are required to assess the impact of treatment lines and ET regimens on survival outcomes. Finally, data were not available within this study to fully define the patient population that may be eligible for chemotherapy according to clinical guidelines (*i.e.*, with rapidly progressive disease or proven endocrine resistance). Therefore conclusions about guideline-adherent therapy decisions were not possible in this study.

Our realistic picture of daily practice offers a good overview of the management of HR+ ABC, such as the choice of palliative treatment and drug selection of ET. To our knowledge, this is the first nation-wide study to identify ET practice patterns for HR+ ABC in China which may provide reference for investigators worldwide and multinational pharmaceutical companies. The results generated from a relatively large patient population could give valuable insight into the actual treatment delivered to the HR+ ABC patients. Previous studies have found that breast cancer characteristics in China displayed distinct features from the USA, including significant younger onset age and higher stage (41) which may influence the choice of treatment in routine practice. Our epidemiological data demonstrated that there are notable differences in physician prescription preferences and accessibility to new drugs of ABC between China and the West, which revealed that China possesses great potential for anti-tumor pharmaceutical market including ET agents and molecular-targeting drugs in combination with ET. This may provide important insight into the strategic layout and market decisions and positioning of new drug research and development for transnational pharmaceutical enterprises.

### Expert Commentary on Management of ABC in China

- Improve the health care system and increase financial support to reduce out-of-pocket medical expenses.
- Increase public awareness and breast cancer screening rate to prevent stage IV disease.
- Accelerate approval of new drugs and improve quality control of generic drugs.
- Improve availability and quality of anticancer therapies for advanced breast cancer.
- Enhance the quality of surgery, radiation, essential medicines, and clinical trials to improve the disease free survival (DFS).
- Expand training of physicians and promote international collaboration on multicenter clinical trials of drugs.

## Data Availability Statement

The original contributions presented in the study are included in the article/**Supplementary Material**. Further inquiries can be directed to the corresponding authors.

## Ethics Statement

This study was approved by the Ethics Committee of Cancer Institution and Hospital, Chinese Academy of Medical Sciences. Patient consent for this study was not required as there were no anticipated risks to the enrolled patients. All data included in this analysis were in aggregate, de-identified information.

## Author Contributions

BX, JW, JF, and YQ were involved in the study design. All authors were responsible for the data collection and case confirmation. YW did the statistical analyses and drafted the manuscript. YH, BX, and JW modified and revised the manuscript. All authors contributed to the article and approved the submitted version.

## Funding

This work was supported by the Investigator-initiated program of the National Cancer Center/National Clinical Research Center for Cancer/Cancer Hospital, Chinese Academy of Medical Sciences and Peking Union Medical College (CH-BC-045).

## Conflict of Interest

The authors declare that the research was conducted in the absence of any commercial or financial relationships that could be construed as a potential conflict of interest.

## Supplementary Material

Supplementary Figure 1 Initial palliative treatment option for HR+ patients according to HER2 status. *ET*, endocrine therapy; *CT*, chemotherapy.

Supplementary Figure 2 Endocrine regimens use by line and age for HR+ABC patients. *SERMs*, selective estrogen receptor modulator; *AIs*, aromatase inhibitors; *OFS*, ovarian function suppression; *mTOR*, mammalian target of rapamycin.
