# Supplementary figures and images for: Endocrine Therapy for Hormone Receptor-Positive Advanced Breast Cancer: A Nation-Wide Multicenter Epidemiological Study in China
Source: Front Oncol. 2021 Feb 11;10:599604. doi: 10.3389/fonc.2020.599604 (PMC7905089; doi:10.3389/fonc.2020.599604)

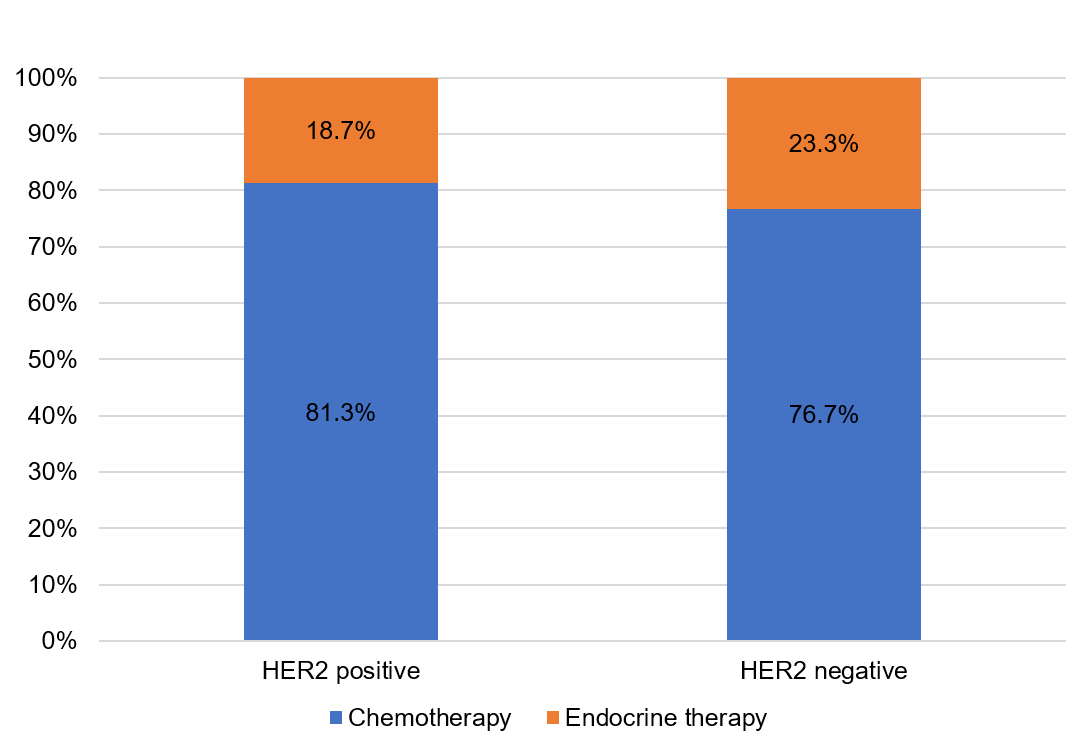

Supplement: Supplementary Figure 1 — Initial palliative treatment option for HR+ patients according to HER2 status. ET, endocrine therapy; CT, chemotherapy. [file Image_1.tif]

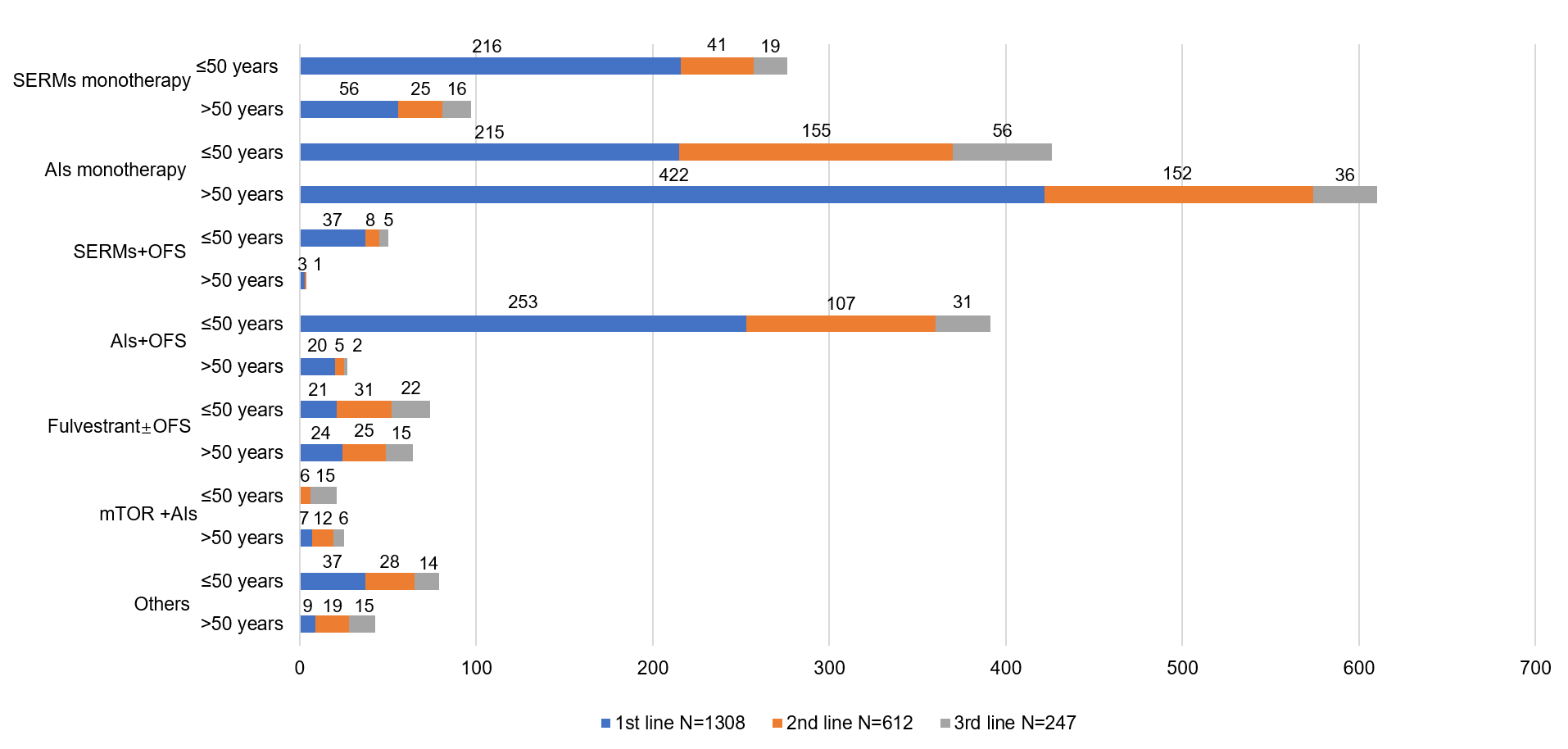

Supplement: Supplementary Figure 2 — Endocrine regimens use by line and age for HR+ABC patients. SERMs, selective estrogen receptor modulator; AIs, aromatase inhibitors; OFS, ovarian function suppression; mTOR, mammalian target of rapamycin. [file Image_2.tif]
